# Persistence of auditory modulation of wind-induced escape behavior in crickets

**DOI:** 10.3389/fphys.2023.1153913

**Published:** 2023-05-09

**Authors:** Anhua Lu, Matasaburo Fukutomi, Hisashi Shidara, Hiroto Ogawa

**Affiliations:** ^1^ Graduate School of Life Science, Hokkaido University, Sapporo, Japan; ^2^ Department of Biology, Washington University in St. Louis, St. Louis, MO, United States; ^3^ Department of Biological Sciences, Faculty of Science, Hokkaido University, Sapporo, Japan; ^4^ Department of Biochemistry, Graduate School of Medicine, Mie University, Tsu, Japan

**Keywords:** escape behavior, cricket (*Gryllus bimaculatus*), multisensory integration, crossmodal interactions, contextual memory, behavioral choice

## Abstract

Animals, including insects, change their innate escape behavior triggered by a specific threat stimulus depending on the environmental context to survive adaptively the predators’ attack. This indicates that additional inputs from sensory organs of different modalities indicating surrounding conditions could affect the neuronal circuit responsible for the escape behavior. Field crickets, *Gryllus bimaculatus*, exhibit an oriented running or jumping escape in response to short air puff detected by the abdominal mechanosensory organ called cerci. Crickets also receive a high-frequency acoustic stimulus by their tympanal organs on their frontal legs, which suggests approaching bats as a predator. We have reported that the crickets modulate their wind-elicited escape running in the moving direction when they are exposed to an acoustic stimulus preceded by the air puff. However, it remains unclear how long the effects of auditory inputs indicating surrounding contexts last after the sound is terminated. In this study, we applied a short pulse (200 ms) of 15-kHz pure tone to the crickets in various intervals before the air-puff stimulus. The sound given 200 or 1000 ms before the air puff biased the wind-elicited escape running backward, like the previous studies using the longer and overlapped sound. But the sounds that started 2000 ms before and simultaneously with the air puff had little effect. In addition, the jumping probability was higher only when the delay of air puff to the sound was 1000 ms. These results suggest that the cricket could retain the auditory memory for at least one second and alter the motion choice and direction of the wind-elicited escape behavior.

## 1 Introduction

Prey animals rely on escape responses to defend themselves against predators’ attacks (Domenici et al., 2011a; b; LeDoux and Daw, 2018). These quick escape responses are not a simple reflex triggered by the presence of an approaching predator, but mediated via complex sensory-motor control, resulting in an action that maximizes the chances of survival for the animal. Many species of prey animals can change their innate escape behavior triggered by a specific threat stimulus depending on the surrounding contexts (Domenici, 2010; Domenici et al., 2011b). For example, the presence of refugees or burrows affects the escape trajectories in various species of prey animals (Hemmi, 2005; Ellard and Eller, 2009; Zani et al., 2009; Kanou et al., 2014). This indicates that additional inputs from sensory organs of different modalities indicating surrounding conditions could affect the neuronal circuits to alter the escape strategies. In other words, multisensory integration in the nervous system may be involved in the context dependency of escape behavior.

Multisensory integration underlies the robust perception and appropriate action selection in various species of animals (Stein et al., 1989; Stein and Stanford, 2008; Ohyama et al., 2015). The temporal relationship among sensory inputs of different modalities is one of the crucial factors for multisensory perception. For example, the temporal coincidence of auditory and visual stimuli enhances the multisensory response in the superior colliculus neurons of cats, which mediates attentive and orientation behavior (Meredith et al., 1987). In humans, a preceding auditory cue improves the directionality of subsequent visual detection (McDonald et al., 2000). The temporal relationship should also play an important role in the multisensory integration for the context dependency of escape behavior because sensory information that informs of the surrounding context needs to be retained for some amount of time. Indeed, previous work shows that a specific timing of visual input increases the response probability of sound-evoked escape in larval zebrafish (Mu et al., 2012). However, it is unclear how the temporal relationship is involved in regulating escape strategies rather than responding or not responding.

Here we address this question by focusing on auditory modulation of wind-elicited escape behavior in field crickets *Gryllus bimaculatus* (Fukutomi et al., 2015; Fukutomi and Ogawa, 2017). Crickets exhibit an escape behavior in response to short air puff detected by the abdominal cercal sensory system (Oe and Ogawa, 2013; Sato et al., 2019). This escape behavior can be modulated by additional sensory inputs such as vision (Kanou et al., 2014), antennal mechanosensation (Ifere et al., 2022), and audition (Fukutomi et al., 2015; Fukutomi and Ogawa, 2017). We have reported that auditory stimuli can modulate the moving direction and response threshold of the wind-elicited escape behavior (Fukutomi et al., 2015; Fukutomi and Ogawa, 2017). This modulation is apparent with a high-frequency sound (15 kHz), which suggests approaching bats’ call and triggers avoidance response during a flight but weakened with a 5-kHz sound corresponding to the carrier frequency of conspecific calling song, suggesting that crickets can alter their escape strategies depending on the acoustic context (Moiseff et al., 1978; Hoy et al., 1989; Pollack, 2015; Fukutomi and Ogawa, 2017). However, it remains unsolved what temporal relationship between these two stimuli is required for the auditory modulation of wind-elicited escape. In addition, crickets change their escape behavior, either running or jumping, depending on the intensity and duration of the air-puff stimuli (Sato et al., 2022a). It is, however, unknown whether this action selection is modulated by auditory input because it had been technically difficult to apply the quantitatively identical acoustic and air-puff stimuli to freely moving crickets from a specific angle. In this study, we used a newly developed servosphere treadmill system (Iwatani et al., 2019) and investigated the effects on the wind-elicited escape behavior in crickets when the high-frequency tone sound was given at various intervals to the air-puff stimulus.

## 2 Materials and methods

### 2.1 Animals

We used a wild-type strain of field crickets (*Gryllus bimaculatus*, Hokudai WT; Watanabe et al., 2018) that were bred in our laboratory. Adult male crickets, less than 14 days after the imaginal molting, were used throughout the experiments. They were reared under 12:12-h light/dark conditions at a constant temperature of 27°C. The guidelines of the Institutional Animal Care and Use Committee of the National University Corporation, Hokkaido University, Japan, specify no requirements for the treatment of insects in experiments.

### 2.2 Preparation and experimental condition

The antennae of the crickets were removed to eliminate the influence of mechanosensory inputs from the antennal organ, which enabled to focus on the interaction between the cercal and auditory systems. All behavioral experiments were conducted during the dark phase of the crickets at room temperature (26°C–28°C) under white LED illumination in a sound-attenuated room (AMC-3525, O’HARA and Co., Ltd., Tokyo, Japan) where anechoic foam sheets (F2-PF, Strider, Toyohashi, Japan) were attached to the ceiling and inner walls.

### 2.3 Treadmill system

**FIGURE 1:**
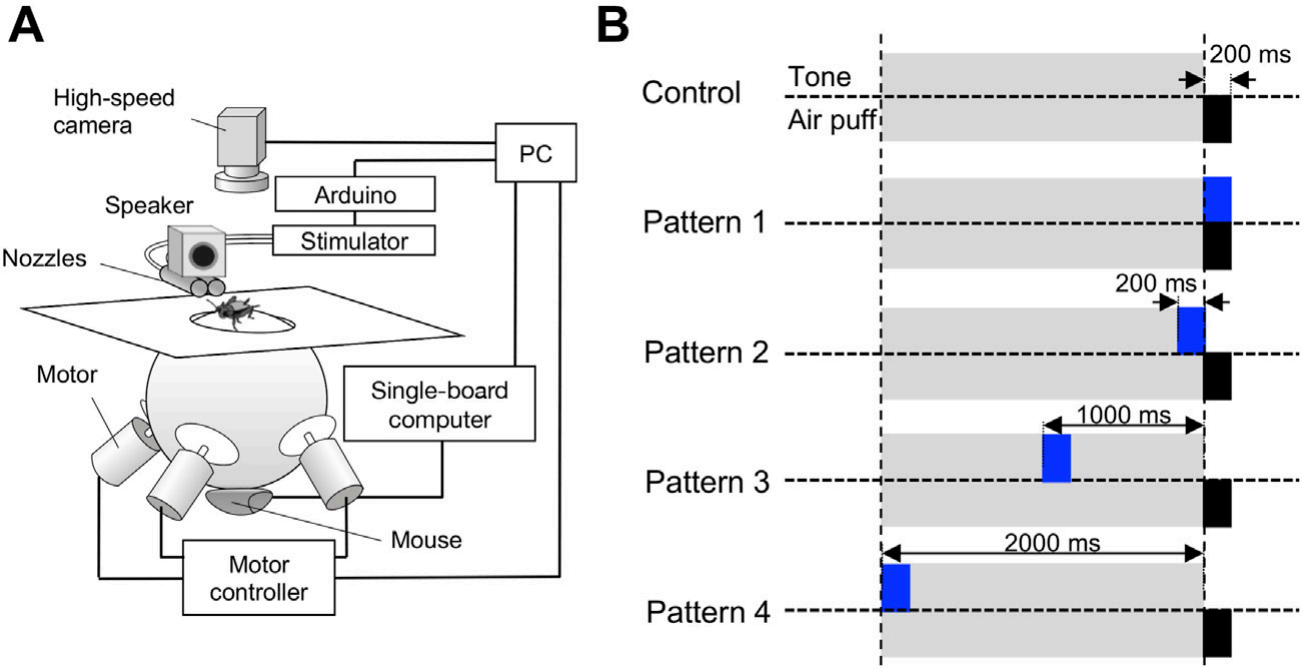
Experimental apparatus and stimulation patterns for behavioral tests. **(A)** Servo-sphere treadmill system for stimulation and monitoring of the freely moving cricket. The air-puff stimulus was applied from the right or left side of the cricket, which was specified by controlling the orientation of the cricket’s body against the air nozzle with the servo-sphere treadmill system that was closed-loop controlled based on the high-speed camera image of the cricket. **(B)** Temporal arrangement of the air-puff and acoustic stimuli for the five different patterns. For the control pattern, only single air-puff stimuli were applied without the acoustic stimulus. For patterns 1-4, a 15-kHz pure tone sound of 200-ms duration was applied from a loudspeaker above the air nozzle, and the air-puff stimulus was initiated 0, 200, 1000, and 2000 ms after the onset of the acoustic stimulus, respectively.

To apply the quantitatively identical auditory and cercal stimuli to the free-moving cricket, we adopted a markerless visual feedback servo-sphere treadmill system (Iwatani et al., 2019). In this system, a Styrofoam ball (ø = 200 mm) was supported by three motor-driven omni-wheels. The treadmill system was installed within a sound-proofed wood box, and its top was covered with a white paper board of 500 mm × 500 mm except for the top 100-mm diameter of the ball. A region of 350 mm × 450 mm, including the ball top and whiteboard, was monitored by a high-speed digital video camera (acA 1920-155 um, BASLER, Ahrensburg, Germany), which was suspended at 800-mm height above the treadmill from a ceiling of the sound-attenuation room (resolution, 1,216 × 1,200 pixels; shutter speed, 1 ms; sampling rate, 160 frames/s, (Figure 1A). Full size of the captured image was used for tracking a cricket and a half size of them was stored for the offline analysis of escape behavior. While an animal was on the treadmill ball, the animal’s location and head orientation were estimated automatically by an image processing technique proposed in a previous report from each captured image (Iwatani, 2021). The treadmill ball was regulated by rotation of the omni-wheels so that the center of the animal’s body and its major axis were always kept at the center at the top of the treadmill ball and to a specific orientation, respectively. This negative feedback allowed the animal moving on the treadmill to be positioned in the specific location and head direction relative to the stimulus nozzle and loudspeaker. This system was feedback-controlled at a rate of 160 times per second. The escape movements caused by the airflow stimulus were recorded by the high-speed camera used for the feedback control of the treadmill. Spontaneous movement of the cricket before the air-current stimulation was detected as the rotation of the Styrofoam ball by an optical mouse placed under the treadmill.

### 2.4 Stimulation

An air-puff stimulus was provided to the stationary cricket by a short puff of nitrogen gas from a plastic nozzle (ø = 15 mm) connected to a pneumatic picopump (PV820, World Precision Instruments, Sarasota FL, USA). For the behavioral experiments using the treadmill system, two air-puff nozzles were arranged in parallel to align their center to the same horizontal plane as the top of the treadmill sphere (Figure 1A). Nozzle ends were placed at 200 mm from the animal. The velocity of the air puffs was controlled at 1.00 m/s, which was measured at the center of the arena with a 405-V1 thermal anemometer (Testo, Yokohama, Japan), by adjusting the delivery pressure of the picopump. The duration of the air-puff stimulus was 200 ms. By changing the cricket’s body axis relative to the stimulus nozzle, the air puffs were applied to the right or left side of the cerci from one of the air nozzles, which was close to the posterior side of the crickets.

The acoustic stimuli were 15-kHz pure tones, synthesized using RPvdsEx software (Tacker Davis Technologies, Alachua FL, USA), and transduced and attenuated using an RM1 processor (TDT). The sounds were calibrated at an average of 70 dB SPL and delivered by a 1.5-inch (3.81 cm) full-range sealed loudspeaker (MM-SPS2, Sanwa Supply, Okayama, Japan), which was located just above the air-puff nozzles at 200 mm away from the animal (Figure 1A).

### 2.5 Stimulation protocols and experimental procedure

To test the cross-modal effects of temporal relationships between the acoustic and air-puff stimuli on the wind-elicited escape behavior, we designed five types of stimulation protocols, referred to as patterns 1-4, in which the air puff was combined with acoustic stimuli, and the control stimulated by the air puff without them (Figure 1B). For the control, only a single air puff of 200-ms duration stimulus was applied without the acoustic stimulation. For the stimulation protocols of patterns 1-4, a 15-kHz pure tone sound of 200-ms duration was started at 0, 200, 1000, or 2000 ms before the air-puff stimulus (Figure 1B). In all stimulation patterns, including the control for the behavioral experiments, a single air-puff stimulus was delivered alternatively from a nozzle to the left or right side of the cricket.

When a cricket was placed on the top of the treadmill, the feedback control of the servosphere was immediately started for the cricket to be positioned at the center of the treadmill and oriented on the left or right side relative to the stimulus nozzle. Every trial, regardless of with or without tone, started only when the cricket kept still for at least 1 s, defined by movement velocity < 10 mm/s measured with the optical mouse under the treadmill sphere. The air-puff stimulus was provided 2 s after the trial started. During these 2 s, the tone sound was played with a specific delay to the air puff. When the cricket moved during these 2 s, the air-puff stimulation was canceled, and the trial restarted after the cricket kept still for at least 1 s. At the onset of air-puff stimulation, the feedback control of the servosphere treadmill was stopped, allowing us to record the cricket’s movement trajectory with the high-speed camera.

The crickets were divided into 4 groups for different stimulation patterns (patterns 1–4). As each experimental group consisted of 15 individuals, 60 crickets were used for the experiments in total. Each individual cricket was exposed to 4 different types of stimulation: control-right, control-left, with sound-right, with sound-left. For “with sound,” the airflow and sound were applied from the same side in any one of the combined stimulation patterns 1–4. The order of stimulation types was randomized, and this randomized stimulation set was repeated 10 times. Thus, 40 trials were conducted in succession for each individual in total. The inter-trial intervals were 1 min or more. The same pattern of the combined stimulation was used for one individual.

### 2.6 Image processing

To compensate for sample-by-sample errors in distance measurements due to angle of view and camera positions, only the area of 350 mm × 450 mm on the opposite side to the stimulus nozzle, which was indicated by four black spots on the white paper board, was cut out from the video image and transformed into a 406 × 522 pixels image. After approximating the contour of the cricket body as an ellipse based on a binarized image, the location and body axis of the cricket were determined by the centroid and the long axis of the ellipse, respectively. The head orientation was set manually on the first image for each sample.

### 2.7 Criteria for the wind-elicited escape responses

The wind-elicited responses of the cricket were analyzed in a manner similar to previous studies (Sato et al., 2017; Sato et al., 2019; Sato et al., 2022a; Sato et al., 2022b). Whether the cricket responded or not was determined based on the translational velocity of its movement. If the translational velocity exceeded 10 mm/s during the period from the stimulus onset to 250 ms after the stimulus onset and was greater in its maximum value than 50 mm/s, the cricket was considered to “respond” to the air current. If the cricket did not begin to move within 250 ms of the response definition period, the trial was considered as “no response.” All the “responding” trials were further categorized into “jumping” or “running” according to leg movements during escape action, which was determined visually for all escaping trials by frame check of the video, as in the previous studies (Sato et al., 2017; Sato et al., 2019; Sato et al., 2022a; Sato et al., 2022b). If all six legs were off the ground simultaneously, that response was defined as “jumping.” If any one of the six legs touched the ground during movement, that response was defined as “running” (see typical running and jumping responses shown in Supplementary Videos S1, S2 of Sato et al., 2022b). The initial response in which both jumping and running movements were observed, for example, the jumping followed by running and the jumping after running, was also defined as “jumping.”

### 2.8 Quantification of escape responses

The selection ratio of running or jumping was calculated as the proportion of responses for all the responding trials. The jumping probability was calculated from the number of jumping responses per total responding trial for each stimulation pattern. The movements in the escape behavior were analyzed for the “initial response” in the responding trials as in previous studies (Oe and Ogawa, 2013; Sato et al., 2017; Sato et al., 2019; Sato et al., 2022a; Sato et al., 2022b). The start of the initial response was defined as when the translational velocity exceeded 10 mm/s for the first time just before reaching 50 mm/s. The finish was defined as when the velocity was less than 10 mm/s for the first time after the response started. Reaction time, maximum movement velocity, and movement distance were measured as metric parameters that characterized the escape movement. The definition of these parameters was the same as those in the previous studies (Oe and Ogawa, 2013; Sato et al., 2017; Sato et al., 2019; Sato et al., 2022a; Sato et al., 2022b). The reaction time was measured as the delay from opening the delivery valve in the picopump to the start of the initial response. The movement distance was measured as the entire path length of the 2-dimensional moving trajectory that was traced for the centroid of the approximated ellipse from the stimulated point to the termination point of the initial response on the video data. The movement direction as the angular parameter was measured as the angle between the body axis at the stimulated location and a line connecting the stimulated location and the response finish location.

### 2.9 Statistical methods

R programming software (ver. 4.0.2, R Development Core Team) was used for the statistical analysis. For analyses of the locomotion parameters measured in the behavioral experiment, such as movement direction, maximum movement velocity, movement distance, and reaction time, to avoid pseudo-replication, we used mean values of the data obtained in the responding trials for each individual as the representative values for the statistical tests. The movement direction is a circular parameter, but the mean angle of it for each individual ranged from 60° to 140°, so this parameter was analyzed as a metric parameter like other locomotion parameters. Prior to the statistical testing of the locomotion parameters, we checked the distribution of the datasets using the Shapiro-Wilk test. As the data of these parameters in the behavioral experiment were normally distributed, we used the paired *t*-test between the control and combined stimulation patterns to assess the significance of the effects on the running response of the acoustic stimulation. Since some individuals showed jumping responses to only one of the control or combined stimulations, we used the unpaired *t*-test to assess the auditory effects on the jumping responses. The Wilcoxon signed-rank test was used to assess the significance of the stimulation condition for the response and jumping probabilities those were not normally distributed.

## 3 Results

### 3.1 Preceding high-frequency sounds increased the choice of jumping response

To investigate how long the impact of the auditory stimuli on the wind-elicited escape behavior lasted, we designed four patterns of the stimulation pattern combining the acoustic stimulus and air puff, which differed in the delay (0, 200, 1000, or 2000 ms) from the onsets of the tone sound to that of the air puff (Figure 1B). The stimulation pattern in which only air puff was applied without the tone sound was used as a control. Crickets exhibit two distinct escape actions, running and jumping, in response to air-puff stimuli (Sato et al., 2017; Sato et al., 2019; Sato et al., 2022a; Sato et al., 2022b), but it is untested whether the auditory stimulus can affect this action selection. The servosphere treadmill system allowed the crickets to escape not only by running but also by jumping in response to the air-puff stimulus, unlike the tethered treadmill system (Oe and Ogawa, 2013; Fukutomi et al., 2015; Fukutomi and Ogawa, 2017). In all stimulation patterns, most of the crickets exhibited wind-elicited escape behaviors, either running or jumping, as reported in the previous study of free-moving crickets (Sato et al., 2017; Sato et al., 2019; Sato et al., 2022a; Sato et al., 2022b).

**FIGURE 2:**
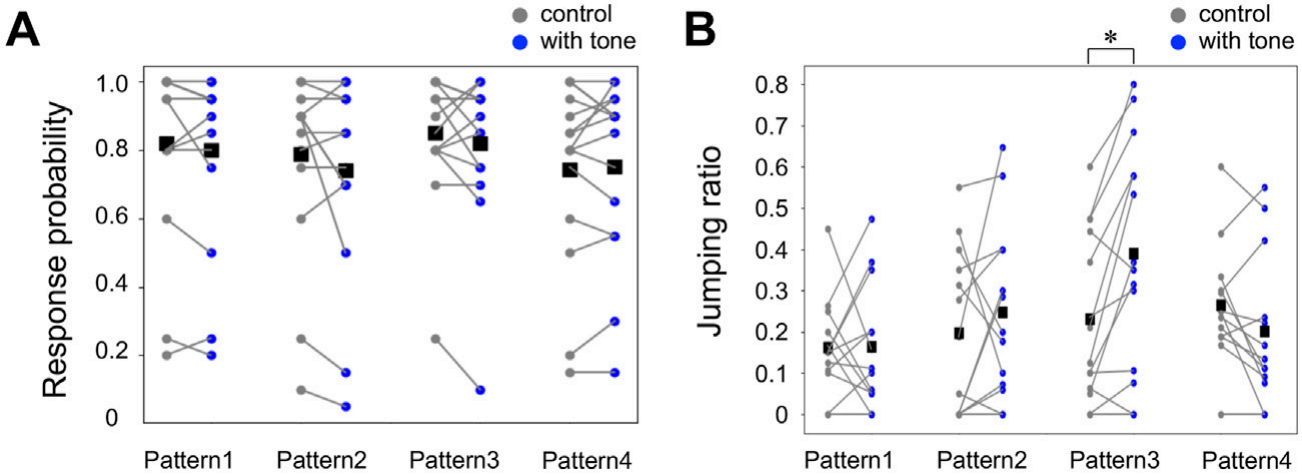
Auditory effects on the response probability and the action selection between running and jumping. **(A, B)** The probability of escape response in all trials **(A)** and the jumping probability in the responding trials **(B)** for the combined stimulation patterns (blue) and control (gray). Small dots connected by lines indicate the mean of the locomotion parameters for each individual. Black squares indicate the average of the data of all individuals. *p < 0.05, Wilcoxon rank-sum test. N = 15 individuals for each combined stimulation pattern.

Firstly, we examined the probability that the crickets exhibited the escape response, including running and jumping. The preceding tone did not modify the probability of responses to the airflow stimuli of the velocity we used in this study (Figure 2A, Supplementary Datasheet S1). The behavioral responses that we recorded were classified into either running or jumping based on the cricket’s leg movements. Then, we compared the selection ratio of the running and jumping in all responding trials among different stimulation patterns (Figure 2B, Supplementary Datasheet S1). In control, jumping was observed in 21% of all responding trials. When the sound stimulus was applied 1000 ms before the air puff in Pattern 3, the jumping ratio to all responding trials was 39%, which was significantly higher than that in the control. In contrast, the jump ratios for other stimulation patterns 1, 2, and 4 were 19%, 25%, and 22%, respectively, and were almost the same as the control. These results indicated that the crickets chose the jumping more frequently in the wind-elicited escape behavior when they heard the high-frequency sound applied 1000-ms before the air-puff stimulus.

### 3.2 Auditory effect on the motor parameters of the escape behavior

**FIGURE 3:**
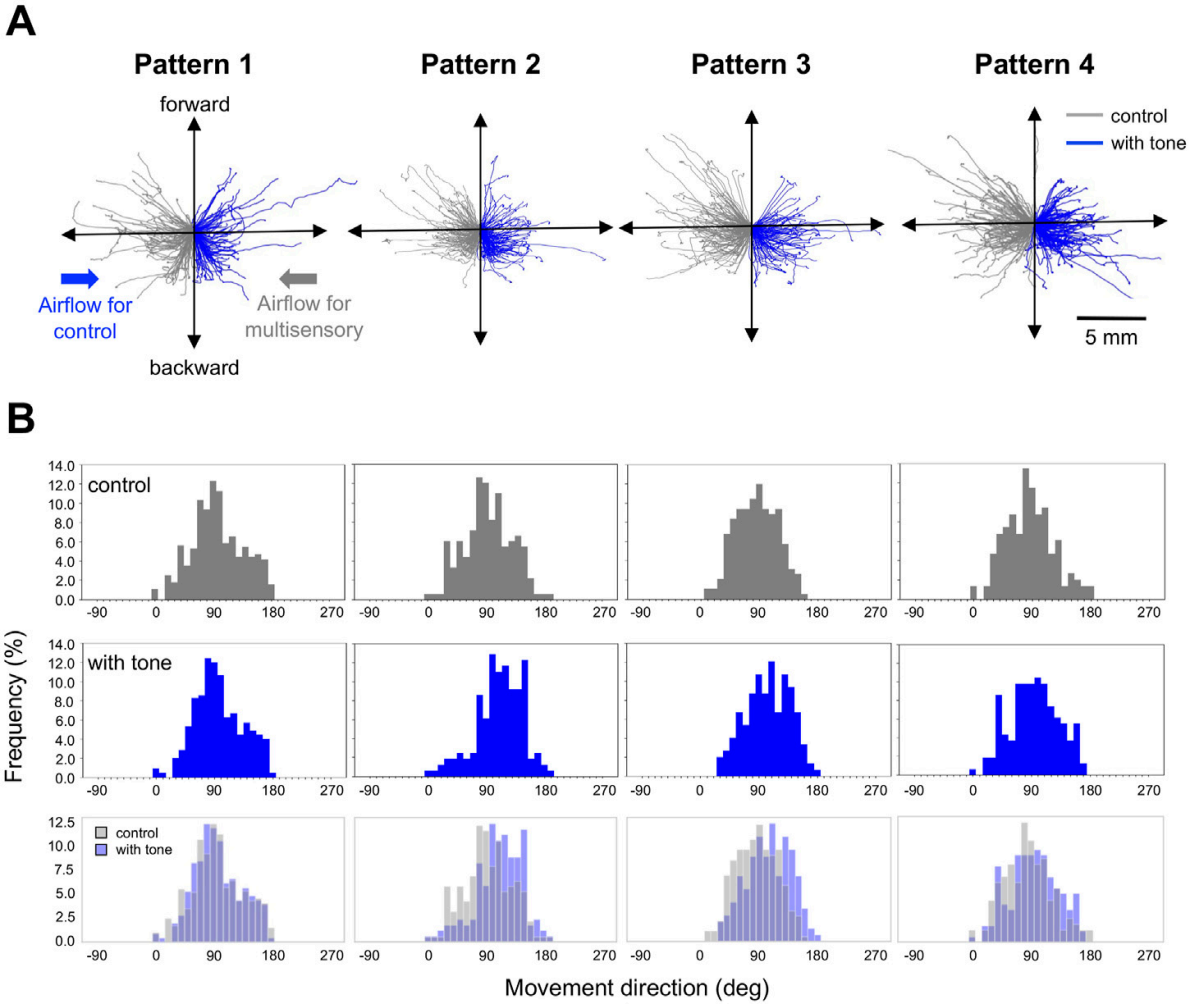
Movements in the running escape responses. **(A)** Typical running trajectories of given individuals in the running response to air puffs combined with and without acoustic stimulus. Gray traces indicate the response in control. Blue traces indicate the response to air-puff combined with tone sound, 0 ms (pattern 1), 200 ms (pattern 2), 1000 ms (pattern 3), and 2000 ms (pattern 4) before the air-puff onset. The trajectories were combined data of the responses to left and right stimuli. The trajectories for the control condition were displayed against the air puff from the right side, and those for the combined stimulation conditions were displayed against the air puff from the left side. **(B)** Distributions of running direction for different stimulation patterns. Gray bars represent the data for controls, and blue bars represent those for patterns 1, 2, 3, and 4. *N* = 15 individuals for control and each acoustic stimulation pattern.

**FIGURE 4:**
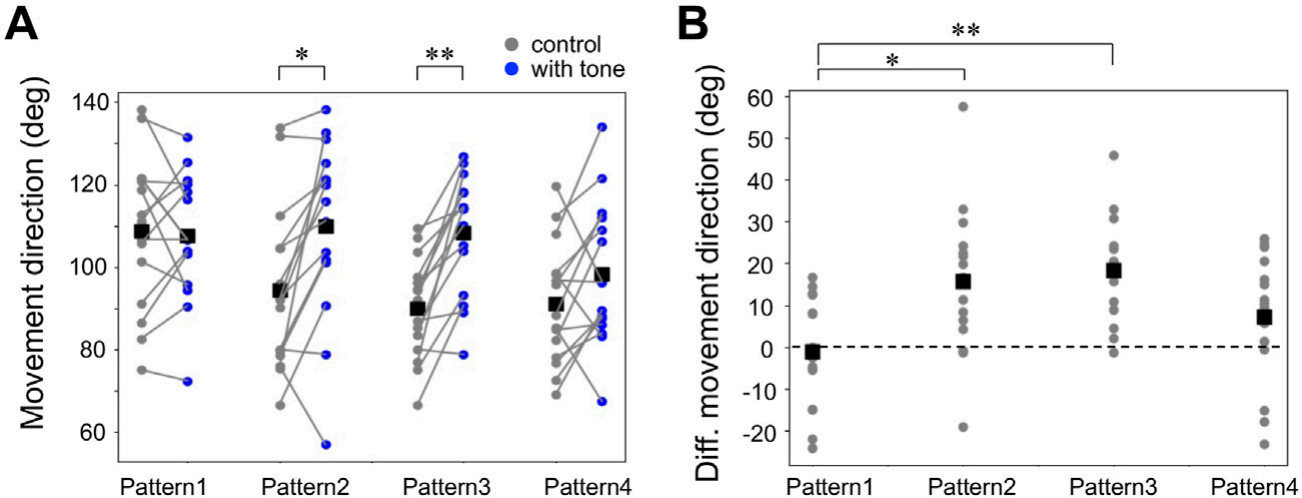
Auditory effects on the movement direction in the running response. **(A)** Movement direction for control (gray) and combined stimulation patterns (blue). Small dots connected by lines indicate the mean angle of the movement directions for each individual. Black squares indicate the average of the data of all individuals. The direction opposite to the air puff is indicated as positive. *p < 0.05, **p < 0.01, paired *t*-test. **(B)** Changes in movement direction by acoustic stimulation. Small gray dots represent the mean of the data of each individual. *p < 0.05, **p < 0.01 unpaired *t*-test with Bonferroni correction. *N* = 15 individuals for each acoustic stimulation pattern.

Next, we focused on the auditory effects of the running escape that have been reported in previous studies on the crickets tethered on an air-lifted treadmill (Fukutomi et al., 2015; Fukutomi and Ogawa, 2017). It has been confirmed that the 15-kHz tone sound we used alone does not induce any locomotion in crickets (Fukutomi and Ogawa, 2017). The running trajectories in all trials responding to the air puff applied from the side of the crickets indicated that they moved toward the side opposite to the stimulus (Figure 3A). When the 15-kHz tone sounds were applied simultaneously with the air puff, referred as Pattern 1, there was no difference in the trajectories compared to the control (gray traces in Figure 3A). In contrast, when the tone sound was given 200–2000 ms before the air puff as in Patterns 2, 3, and 4, the trajectories seemed to be extended more backward (Figure 3A). The distributions of running direction, therefore, for the stimulation patterns 2-4 were likely shifted to backward compared to that for control (Figure 3B). The statical analysis indicated that the running direction for the pattern 2 and 3, in which the air puffs were applied 200 ms and 1000 ms after the sound started, was significantly greater than that for the control (Figure 4A, Supplementary Datasheet S1). In contrast, there was no significant difference in the running direction for patterns 1 and 4 (Figure 4A), indicating that the sounds given 0 ms or 2000 ms before the air puff had little effect on the movement direction in the running escape. These results suggest that the auditory modulation of movement direction requires the tone sounds preceding the air puff, not simultaneous to it. In addition, the magnitude of the auditory effect on the movement direction was measured as the difference between the control condition without acoustic stimulus and the test condition with tone and was examined for delay times from acoustic to airflow stimuli (Figure 4B, Supplementary Datasheet S1). The high-frequency sound starting 1000 ms before the airflow onset seemed to be still as effective as that immediately before the airflow, even though there was a silent period of 800 ms before the airflow onset. And the sound given 2000 ms before the airflow onset (the silent period between the sound and airflow was 1800 ms) appeared less effective. These results indicated that the auditory effect on the movement direction lasted at least 800 ms after the end of the sound stimulus but was not maintained for 1800 ms.

**FIGURE 5:**
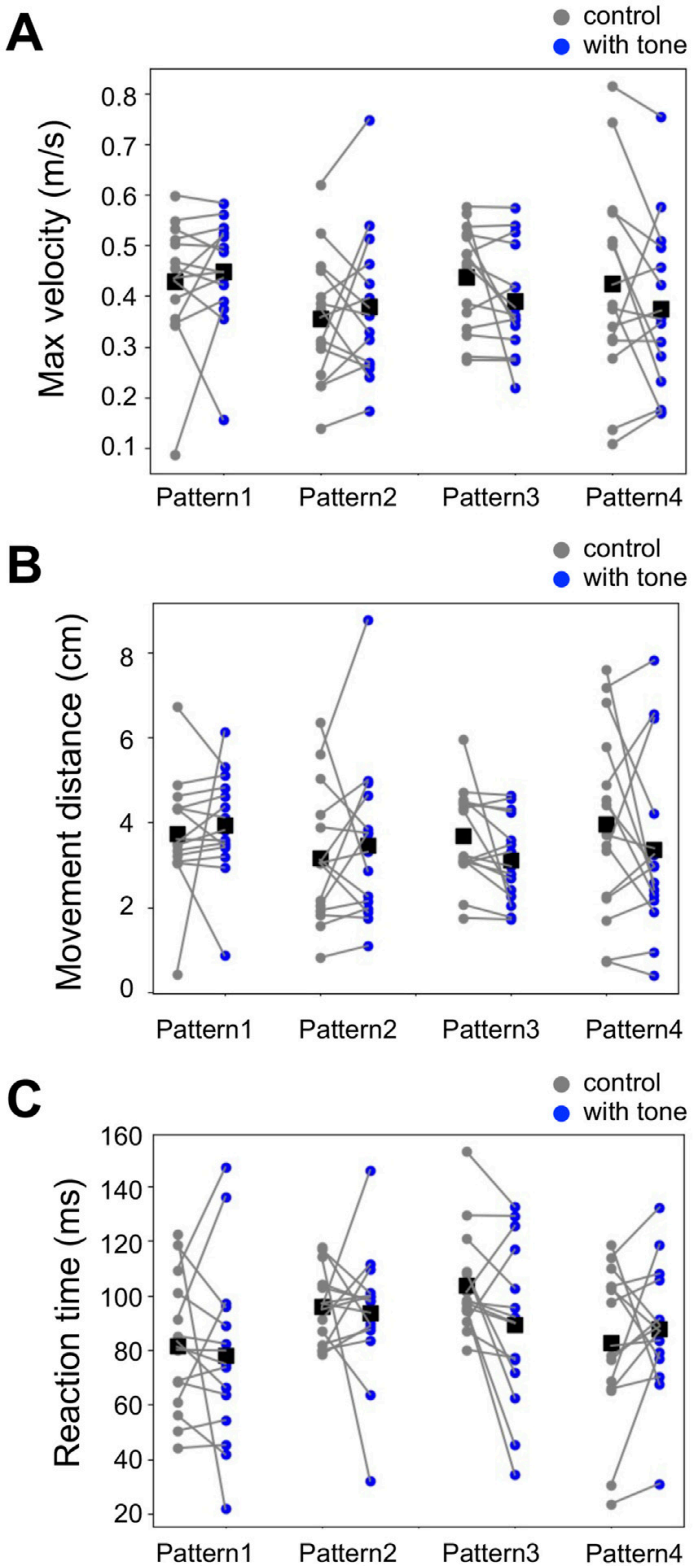
No effects of acoustic stimulation on other locomotion parameters for the running response. **(A–C)** Maximum movement velocity **(A)**, movement distance **(B)**, and reaction time **(C)** in the escape running responses for the different patterns of combined stimulation (blue) and control (gray). Small dots connected by lines indicate the mean of the locomotion parameters for each individual. Black squares indicate the average of the data of all individuals. There was no significant difference in these parameters between combined stimulation patterns (1–4) and control (paired *t*-test). *N* = 15 individuals for control and each acoustic stimulation pattern.

Although the previous works did not detect auditory effects on the other locomotion parameters, it was possible that the different temporal relationships of tone and air puff could modulate them (Fukutomi et al., 2015; Fukutomi and Ogawa, 2017). Then, we examined the auditory modulation of locomotion parameters in the running escape, including the maximum movement velocity, the movement distance, and the reaction time (Figure 5, Supplementary Datasheet S1). The measured values of these parameters varied among the different groups of crickets but were not significantly different for any stimulation patterns compared to the controls. This result indicated that these parameters are not affected by sound regardless of the temporal relationship.

**FIGURE 6:**
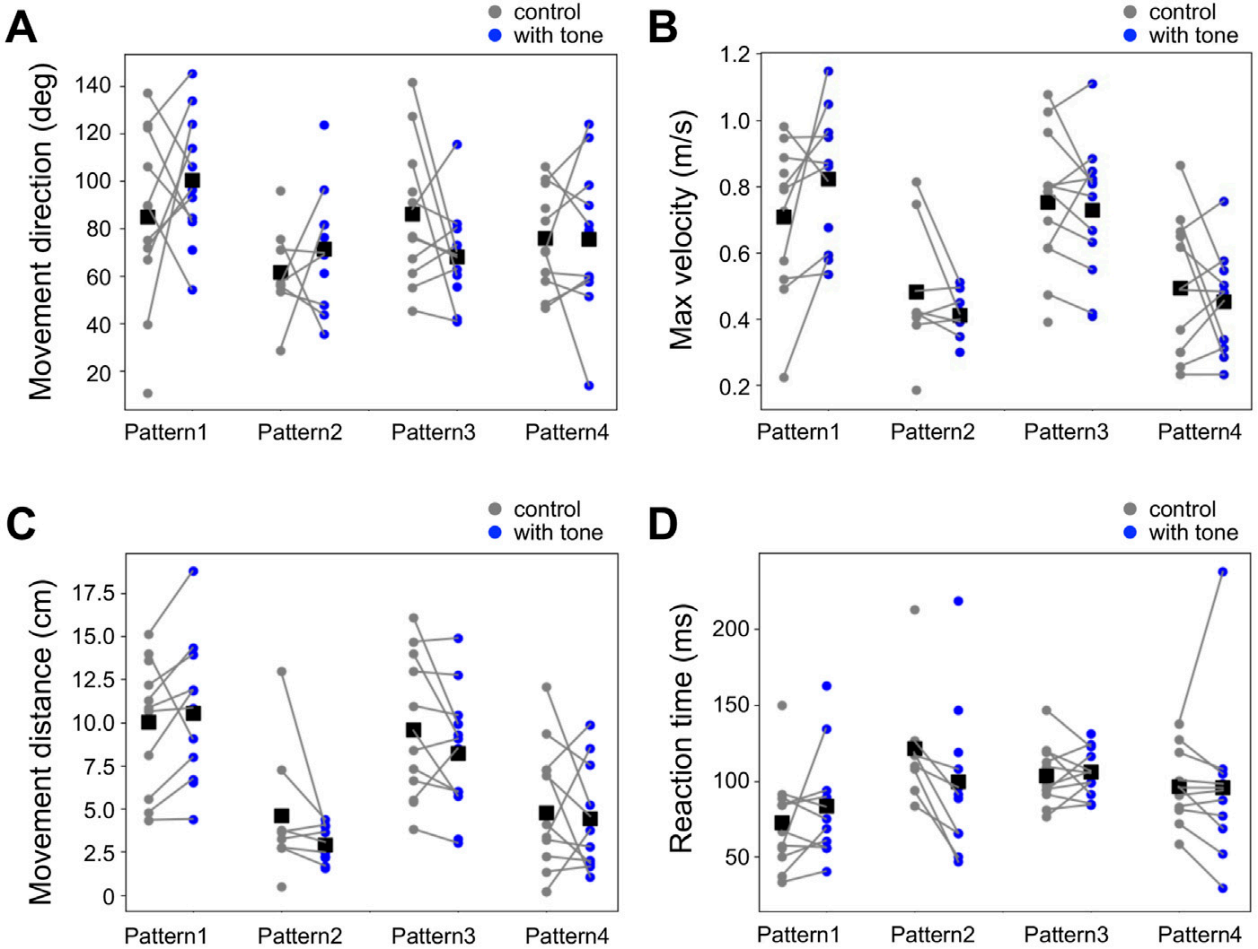
No effects of acoustic stimulation on the locomotion in the jumping response. **(A–D)** Movement direction **(A)**, maximum movement velocity **(B)**, movement distance **(C)**, and reaction time **(D)** in the escape jumping responses for the different patterns of combined stimulation (blue) and control (gray). Small dots connected by lines indicate the mean of the locomotion parameters for each individual. Black squares indicate the average of the data of all individuals. There was no significant difference in these parameters between combined stimulation patterns (1–4) and control (unpaired *t*-test). *N* = 11 and 11 (pattern 1), 8 and 11 (pattern 2), 12 and 12 (pattern 3), and 12 and 11 (pattern 4) individuals for control and each combined stimulation, respectively.

Then, we also investigated the auditory modulation of the locomotion parameters in the jumping response, which were movement direction, movement distance, maximum velocity, and reaction time (Figure 6, Supplementary Datasheet S1). As in the running response, there was no significant difference in any of the parameters of the running response between the control and combined stimulation for all 4 patterns. Taken together, the high-frequency sound applied before the air-puff stimulus did not affect locomotor parameters in both running and jumping escape.

## 4 Discussion

### 4.1 Auditory effect on the behavioral choice

An identical stimulus does not always cause animals to engage in the same behavior. They determine the appropriate response based on the situation, the external stimulus, and their internal state. Crickets exhibit several escape actions, including running and jumping in response to a short air current detected as a predator approaches (Tauber and Camhi, 1995; Dupuy et al., 2011). Recently our study of freely moving crickets in an experimental arena revealed that crickets change the behavioral choice in the wind-elicited escape behavior, either running or jumping, according to the airflow speed and duration (Sato et al., 2022a). Using quantitatively identical acoustic stimulation with the servosphere treadmill system, we succeeded in examining the auditory effect on this behavioral choice in the freely moving crickets. Our results showed that the high-frequency sound applied 1000 ms before the air-puff stimulus increased the choice of jumping. This suggests that the additional auditory input could bias the decision of running or jumping. In addition, unlike the auditory effect on the running direction, this effect on the behavioral choice was not observed when the acoustic stimulus was given 200 ms before the air puff. This indicates that 200 ms is too short for crickets to reflect the auditory information of the context in the behavioral choice.

The behavioral choice includes not only running or jumping but also escaping or not. Previous works showed that the preceding sound elevated the response threshold, i.e., decreased the response probability of the wind-elicited escape (Fukutomi et al., 2015; Fukutomi and Ogawa, 2017). However, we could not find the auditory effect on response probability from the present results. This may have been due to a difference in the airflow intensity used. In this study, to examine the behavioral choice between jumping and running, we used large-velocity air-puff stimuli (1.0 m/s). The response probability curve against air-puff velocity shows that response probability is saturated at around 1.1 m/s (Fukutomi et al., 2015). Thus, it may be difficult to detect the auditory effect on the response probability at that velocity. The lower velocity of the air-puff stimulus should be used to detect the auditory modulation of response probability.

### 4.2 Auditory effects on the running direction lasted at least 1 s

Directional control is one of the most important aspects of the initial phase in escape behavior (Domenici et al., 2011a; b; Nair et al., 2017; Kimura and Kawabata, 2018). Crickets can control the direction of their wind-induced escape according to the angle of the stimulus, as in the escape behaviors of flies and cockroaches (Domenici et al., 2008; Card, 2012). Crickets flee in the opposite direction from which the air-puff stimulus comes, as in the control condition observed in our experiments (Oe and Ogawa, 2013; Sato et al., 2019). The previous study reported that crickets moved more backward in response to the air puff from their side when they heard a high-frequency sound for 800 ms before that threat stimulus (Fukutomi et al., 2015). This modulation is considered to be a behavioral modulation adapting to the presence of echolocating bats, which are a predator of crickets (Fukutomi and Ogawa, 2017). In these previous studies, however, a final part of the preceding tone sound temporally overlapped with the air-puff stimulus. Therefore, it has been unknown whether the temporal coincidence of the acoustic and air-puff stimuli is necessary for the modulation of the escape behavior. In other words, it remains unclear whether the acoustic stimulus terminating before the air-puff stimulus also modulates the escape behavior. If so, how long does the auditory effect last? The present results indicated that the running direction was biased backward when the 200-ms sound pulse was applied 200 or 1000 ms before the air-puff stimulus started. In contrast, the acoustic stimuli that were given simultaneously or 2000 ms before the air puff had little impact on the escape direction. These results demonstrated that preceding auditory inputs rather than simultaneous ones with the air-puff stimulus were necessary for the behavioral modulation.

Interestingly, even when the sound pulse of 200-ms duration was applied 1000-ms before the air-puff stimulus, it also biased the running direction backward. This result also suggested that the acoustic contextual information could be retained for at least 800 ms after the loss of auditory inputs. Temporal relationships between conditioning stimulus (CS) and unconditioned stimulus (US) were crucial rules for classical conditioning to induce associative learning (Mazur, 2006; Mazur, 2012). In classical eye-blink conditioning in mammals, successful learning requires that the sound stimulus as CS precedes the air puff as US, and that simultaneous exposure to both CS and US does not induce the learning. That is because it takes some time for the animals to recognize the CS and to associate the CS with the following US. Probably, this reason accounts for our results that auditory modulation of the running direction required precursor time. The neurophysiological study on eye-blink conditioning in rabbits reported that rostral medial prefrontal cortex (rmPFC) neurons detect CS-US time intervals with dominant firing peaks (Caro-Martín et al., 2015). In the cricket brain, LN3 neuron has been identified as a coincidence detector to recognize the interval of an acoustic pulse of a male’s calling song (∼20 ms) (Schöneich et al., 2015), although the interval is much shorter than 800 ms. Future studies will explore what neural mechanisms could perceive the auditory context and sustain that “memory” to modulate wind-elicited escape behavior.

## 5 Conclusion

Crickets modulate wind-elicited escape behavior depending on acoustic context mediated by cross-modal integration (Fukutomi et al., 2015; Fukutomi and Ogawa, 2017). Here we showed that the auditory modulations of movement direction and jumping probability require preceding auditory input but not simultaneous input. Furthermore, the effect of this contextual information was found to persist for at least 0.8 s after the acoustic stimulus ceased. Future works will examine what neural mechanism supports the persistence of contextual information that influence the behavior.

## Data Availability

The raw data supporting the conclusion of this article will be made available by the authors, without undue reservation.

## References

[B1] Card G. M. (2012). Escape behaviors in insects. Curr. Opin. Neurobio. 22, 180–186. 10.1016/j.conb.2011.12.009 22226514

[B2] Caro-Martín C. Leal-Campanario R. Sánchez-Campusano R. Delgado-García J. M. Gruart A. (2015). A variable oscillator underlies the measurement of time intervals in the rostral medial prefrontal cortex during classical eyeblink conditioning in rabbits. J. Neurosci. 35, 14809–14821. 10.1523/JNEUROSCI.2285-15.2015 26538651 PMC6605228

[B3] Domenici P. Turesson H. Brodersen J. Brönmark C. (2008). Predator-induced morphology enhances escape locomotion in crucian carp. Proc. R. Soc. B 275, 195–201. 10.1098/rspb.2007.1088 PMC2596180 17971327

[B4] Domenici P. Blagburn J. M. Bacon J. P. (2011a). Animal escapology I: Theoretical issues and emerging trends in escape trajectories. J. Exp. Biol. 214, 2463–2473. 10.1242/jeb.029652 21753039 PMC4495464

[B5] Domenici P. Blagburn J. M. Bacon J. P. (2011b). Animal escapology II: Escape trajectory case studies. J. Exp. Biol. 214, 2474–2494. 10.1242/jeb.053801 21753040 PMC3135389

[B6] Domenici P. (2010). Context-dependent variability in the components of fish escape response: Integrating locomotor performance and behavior. J. Exp. Zool. A Ecol. Genet. Physiol. 313, 59–79. 10.1002/jez.580 20073047

[B7] Dupuy F. Casas J. Body M. Lazzari C. R. (2011). Danger detection and escape behaviour in wood crickets. J. Insect Physiol. 57, 865–871. 10.1016/j.jinsphys.2011.03.020 21439965

[B8] Ellard C. G. Eller M. C. (2009). Spatial cognition in the gerbil: Computing optimal escape routes from visual threats. Anim. Cogn. 12, 333–345. 10.1007/s10071-008-0193-9 18956215

[B9] Fukutomi M. Ogawa H. (2017). Crickets alter wind-elicited escape strategies depending on acoustic context. Sci. Rep. 7, 15158. 10.1038/s41598-017-15276-x 29123249 PMC5680309

[B10] Fukutomi M. Someya M. Ogawa H. (2015). Auditory modulation of wind-elicited walking behavior in the cricket *Gryllus bimaculatus* . J. Exp. Biol. 218, 3968–3977. 10.1242/jeb.128751 26519512

[B11] Hemmi J. M. (2005). Predator avoidance in fiddler crabs: 2. The visual cues. Anim. Behav. 69, 615–625. 10.1016/j.anbehav.2004.06.019

[B12] Hoy R. Nolen T. Brodfuehrer P. (1989). The neuroethology of acoustic startle and escape in flying insects. J. Exp. Biol. 146, 287–306. 10.1242/jeb.146.1.287 2689567

[B13] Ifere N. O. Shidara H. Sato N. Ogawa H. (2022). Spatial perception mediated by insect antennal mechanosensory system. J. Exp. Biol. 225, jeb243276. 10.1242/jeb.243276 35072207 PMC8920036

[B14] Iwatani Y. Ogawa H. Shidara H. Sakura M. Sato T. Hojo M. K. (2019). Markerless visual servo control of a servosphere for behavior observation of a variety of wandering animals. Adv. Robot. 33, 183–194. 10.1080/01691864.2019.1570334

[B15] Iwatani Y. (2021). “High-speed servosphere,” in Proceedings of IEEE/SICE International Symposium on System Integration, Iwaki, Fukushima, Japan, January 2021 (IEEE), 613–618.

[B16] Kanou M. Matsuyama A. Takuwa H. (2014). Effects of visual information on wind-evoked escape behavior of the cricket, *Gryllus bimaculatus* . Zool. Sci. 31, 559–564. 10.2108/zs130218 25186926

[B17] Kimura H. Kawabata Y. (2018). Effect of initial body orientation on escape probability of prey fish escaping from predators. Biol. Open 7, bio023812. 10.1242/bio.023812 29945875 PMC6078344

[B18] LeDoux J. Daw N. D. (2018). Surviving threats: Neural circuit and computational implications of a new taxonomy of defensive behaviour. Nat. Rev. Neurosci. 19, 269–282. 10.1038/nrn.2018.22 29593300

[B19] Mazur J. E. (2006). Learning and behavior. 6th ed. Prentice-Hall, Englewood Cliffs, NJ: Pearson Education, Inc.

[B20] Mazur J. E. (2012). Learning and behavior: Instructor’s review copy. 7th ed. New York: Psychology Press.

[B21] McDonald J. J. Teder-Sälejärvi W. A. Hillyard S. A. (2000). Involuntary orienting to sound improves visual perception. Nature 407, 906–908. 10.1038/35038085 11057669

[B22] Meredith M. A. Nemitz J. W. Stein B. E. (1987). Determinants of multisensory integration in superior colliculus neurons. I. Temporal factors. J. Neurosci. 7, 3215–3229. 10.1523/JNEUROSCI.07-10-03215.1987 3668625 PMC6569162

[B23] Moiseff A. Pollack G. S. Hoy R. R. (1978). Steering responses of flying crickets to sound and ultrasound: Mate attraction and predator avoidance. Proc. Nat. Acad. Sci. U. S. A. 75, 4052–4056. 10.1073/pnas.75.8.4052 PMC392929 16592556

[B24] Mu Y. Li X.-Q. Zhang B. Du J.-L. (2012). Visual input modulates audiomotor function via hypothalamic dopaminergic neurons through a cooperative mechanism. Neuron 75, 688–699. 10.1016/j.neuron.2012.05.035 22920259

[B25] Nair A. Changsing K. Stewart W. J. McHenry M. J. (2017). Fish prey change strategy with the direction of a threat. Proc. R. Soc. B 284, 20170393. 10.1098/rspb.2017.0393 PMC5489722 28637854

[B26] Oe M. Ogawa H. (2013). Neural basis of stimulus-angle-dependent motor control of wind-elicited walking behavior in the cricket *Gryllus bimaculatus* . PLoS One 8, e80184. 10.1371/journal.pone.0080184 24244644 PMC3828193

[B27] Ohyama T. Schneider-Mizell C. M. Fetter R. D. Aleman J. V. Franconville R. Rivera-Alba M. (2015). A multilevel multimodal circuit enhances action selection in Drosophila. Nature 520, 633–639. 10.1038/nature14297 25896325

[B28] Pollack G. S. (2015). Neurobiology of acoustically mediated predator detection. J. Comp. Physiol. A 201, 99–109. 10.1007/s00359-014-0948-5 25305136

[B29] Sato N. Shidara H. Ogawa H. (2017). Post-molting development of wind-elicited escape behavior in the cricket. J. Insect Physiol. 103, 36–46. 10.1016/j.jinsphys.2017.10.003 29030316

[B30] Sato N. Shidara H. Ogawa H. (2019). Trade-off between motor performance and behavioural flexibility in the action selection of cricket escape behaviour. Sci. Rep. 9, 18112. 10.1038/s41598-019-54555-7 31792301 PMC6889515

[B31] Sato N. Shidara H. Ogawa H. (2022a). Action selection based on multiple-stimulus aspects in wind-elicited escape behavior of crickets. Heliyon 8, e08800. 10.1016/j.heliyon.2022.e08800 35111985 PMC8790502

[B32] Sato N. Shidara H. Kamo S. Ogawa H. (2022b). Roles of neural communication between the brain and thoracic ganglia in the selection and regulation of the cricket escape behavior. J. Insect Physiol. 139, 104381. 10.1016/j.jinsphys.2022.104381 35305989

[B33] Schöneich S. Kostarakos K. Hedwig B. (2015). An auditory feature detection circuit for sound pattern recognition. Sci. Adv. 1, e1500325. 10.1126/sciadv.1500325 26601259 PMC4643773

[B34] Stein B. E. Stanford T. R. (2008). Multisensory integration: Current issues from the perspective of the single neuron. Nat. Rev. Neurosci. 9, 255–266. 10.1038/nrn2331 18354398

[B35] Stein B. E. Meredith M. A. Huneycutt W. S. McDade L. (1989). Behavioral indices of multisensory integration: Orientation to visual cues is affected by auditory stimuli. J. Cogn. Neurosci. 1, 12–24. 10.1162/jocn.1989.1.1.12 23968407

[B36] Tauber E. R. A. N. Camhi J. (1995). The wind-evoked escape behavior of the cricket *Gryllus bimaculatus*: Integration of behavioral elements. J. Exp. Biol. 198, 1895–1907. 10.1242/jeb.198.9.1895 9319804

[B37] Watanabe T. Ugajin A. Aonuma H. (2018). Immediate-early promoter-driven transgenic reporter system for neuroethological research in a hemimetabolous insect. eNeuro 5, e0061. 10.1523/ENEURO.0061-18.2018 PMC6140108 30225346

[B38] Zani P. A. Jones T. D. Neuhaus R. A. Milgrom J. E. (2009). Effect of refuge distance on escape behavior of side-blotched lizards (*Uta stansburiana*). Can. J. Zool. 87, 407–414. 10.1139/z09-029

